# The status and comparison of ovarian reserve between fertile and infertile healthy Chinese women of reproductive age

**DOI:** 10.1097/MD.0000000000025361

**Published:** 2021-04-30

**Authors:** Shan-Jie Zhou, Tie-Cheng Sun, Ling-Li Song, Ming Yang, Xin-Ping Sun, Li Tian

**Affiliations:** a Reproductive Medicine Center, Department of Gynecology and Obstetrics; b Department of Clinical Laboratory, Peking University International Hospital, Beijing, China.

**Keywords:** anti-Müllerian hormone, antral follicle count, fertility, infertility, ovarian aging, ovarian reserve

## Abstract

We aimed to investigate ovarian reserve status, and explore differences in ovarian reserve between fertile and infertile healthy Chinese women of reproductive age.

We recruited 442 fertile women aged 23 to 49 years (mean: 35.22 ± 4.91 years) as subjects, and 196 infertile women aged 23 to 46 years (mean: 32.34 ± 4.34 years) as controls. For all participants, a number of parameters were tested on days 2 to 4 of a spontaneous cycle, including basal serum follicle-stimulating hormone (FSH), estradiol (E2), luteinizing hormone (LH), total testosterone, anti-Müllerian hormone (AMH), ovarian response prediction index (ORPI), and antral follicle count (AFC).

There were significant differences in terms of AFC, serum AMH levels, and ORPI among subject subgroups (10.58 ± 5.80; 2.533 ± 2.146 ng/mL; 1.28 ± 1.87; respectively), and among control subgroups (12.44 ± 5.69; 3.189 ± 2.551 ng/mL; 1.88 ± 2.68; respectively) (*P* < .01 for all). For both subjects and controls, AFC, AMH levels, and ORPI decreased gradually with increasing age, and presented with similar age-related trends; there were positive correlations between AMH and AFC (*P* < .001), and negative correlations between age and AFC, AMH, ORPI (*P* < .05 for all). There was a significant difference in age (*P* < .001), serum E2 (*P* < .01), and AMH (*P* < .01) levels between subjects and controls; however, when controlling for confounding factors (age, body mass index, total testosterone, and LH), we found no differences between the 2 groups with regards to the serum levels of AMH, FSH, E2, and AFC (*P* > .05 for all). Moreover, receiver operating characteristic curve analysis indicated that the significant variables of subjects and controls for evaluating ovarian reserve included age, AMH and ORPI, and ORPI was more valuable than other variables.

A diminished ovarian reserve was one of the manifestations caused by female aging. When confounding factors were controlled for, we found no differences in ovarian reserve when compared between fertile and infertile women, and no correlation with infertility.

## 1 Introduction

All females, particularly those of advanced aged, show a close association between ovarian reserve and the fertilization of sufficient healthy eggs with spermatozoa, impregnation, and the delivery of a healthy child. Thus, the evaluation of ovarian reserve is valuable when predicting female fertility, and to formulate appropriate treatment strategies for infertile women. The combination of advanced female age and diminished ovarian reserve inevitably results in a reduction in pregnancy rates. Many biological indicators have been used to evaluate ovarian reserve and predict ovarian aging, including anti-Müllerian hormone (AMH), antral follicle count (AFC), ovarian volume, follicle-stimulating hormone (FSH), estradiol (E2), inhibin B, and the ovarian response prediction index (ORPI). Moreover, AFC and AMH are known to be the most appropriate follicular markers to best reflect the ovarian reserve; serum AMH was the first marker shown to decrease in association with a decline in ovarian reserve.^[1,2]^

Although many studies have been carried out on serum AMH levels and AFC, there is still some debate relating to differences in these variables between fertile and infertile women. Several studies^[3–5]^ have indicated that infertile women have an ovarian reserve, and similar AFC and AMH levels, that are similar to those of fertile women across all age categories. Infertile patients have also been shown to have similar AMH levels, a similar prevalence of low AMH levels, and a similar AFC, as controls with no history of infertility.^[3]^ Another study indicated that AMH, AFC, and AMH/AFC ovarian reserve indices, did not differ when compared between infertile women and community-based controls.^[5]^ However, other studies relating to ovarian reserve^[6–8]^ reported different views. For example, 2 studies found that there was a significant difference in mean AMH and AFC, but no difference in FSH levels when compared between a nulliparous and a multiparous group,^[6]^ moreover, a significant difference was detected in the mean FSH and AMH of age-matched fertile and infertile women.^[7]^ Another paper stated that AFC and median AMH concentrations were significantly lower in a group of patients with unexplained infertility than a group of patients diagnosed with male factor infertility.^[8]^ Collectively, the results of these previous studies create a conundrum and have created significant debate with regards to the status of ovarian reserve when compared between fertile and infertile women.

In the present study, we enrolled 442 fertile and child-bearing women, along with 196 infertile women. Using these study cohorts, we investigated ovarian reserve status, and explored differences in ovarian reserve between the fertile and infertile healthy Chinese women.

## 2 Materials and methods

### 2.1 Subjects and study group

The study cohort consisted of 442 community-based women aged 23 to 49 years, who had experienced natural pregnancies and childbirth, enrolled between May 2017 and January 2018. In addition, we simultaneously enrolled 196 infertile women aged 23 to 46 years, who visited our fertility clinic to seek assessments of individual fertility and ovarian reserve, as controls. Subjects and controls were determined to have regular menstrual cycles, possessed 2 ovaries, had no history of ovarian surgery, no severe endometriosis, and no evidence of endocrine disorders. Women were excluded if they used hormonal contraceptives, and had any autoimmune, genetic, or iatrogenic conditions (autoimmune endocrinopathies, radiation therapy, or pelvic surgery), as these factors have been shown to alter the serum profiles of reproductive hormone and AMH. Subjects and controls were stratified into the following age groups: 23 to 29 years (Group 1), 30 to 34 years (Group 2), 35 to 39 years (Group 3), and 40 to 49 years (Group 4).

### 2.2 Measurement of reproductive hormones and AMH

Blood samples were obtained by venipuncture at 7:30 am to 10:00 am on days 2 to 4 of a spontaneous natural cycle. The samples were then used to determine the basal serum levels of FSH, luteinizing hormone (LH), E2, total testosterone (TT), prolactin (using commercial kits and electrochemiluminescence assays available from Abbott Ireland Diagnostics Division Lisnamuck, Longford Co., Longford, Ireland), and AMH (using a commercial kit and electrochemiluminescence assays from Roche Diagnostics GmbH, Mannheim, Germany). The sensitivity of the AMH kit was 0.010 ng/mL, the mean intra- and inter-assay coefficients of variations for AMH were 3.41% and 1.30%, respectively.

### 2.3 The measurement of AFC

Experienced and qualified sonographers performed ultrasonographic evaluations for all subjects and controls during days 2 to 4 of a spontaneous natural cycle using a two-dimensional transvaginal probe operating at a frequency of 9 MHz (HD11 XE, Philips Ultrasound, Inc., Bothell, WA). The total number of AFCs with a diameter 2 to 9 mm was determined in 2 ovaries.

### 2.4 Calculation of body mass index, FSH/LH ratio, and ORPI

Height and weight data were used to calculate body mass index (BMI) using the following equation: weight (kg)/(height × height) (m^2^). Serum FSH and LH concentrations were used to calculate the FSH/LH ratio. The ORPI was defined by the following equation: ORPI = (AMH × AFC)/age.^[9]^

### 2.5 Statistical analysis

Microsoft Excel 2016 software (Microsoft Corporation, Redmond, WA) and SPSS 21.0 (IBM Corporation, New York) were used for all statistical analysis. Data are presented as mean ± standard deviation, as calculated for all subjects, controls, and each age subgroup. One-way analysis of variance (ANOVA) was used to investigate differences in variables between different groups. Multivariate analysis of covariance (ANCOVA/MANCOVA) was further used to investigate differences in variables between subjects and controls after controlling for confounding factors. Pearson's correlation analysis was then used to investigate correlations between different variables. Receiver operating characteristic curve (ROC curve) analysis was used to analyze the predictive accuracy of variables, and to calculate the area under the curve (AUC), and the cut-off values and corresponding sensitivity and specificity. The *Z* test was used to assess the differences between the AUC of different parameters. Tests were considered to be statistically significant if *P* < .05.

## 3 Results

### 3.1 The data of key variables related to ovarian reserve

Clinical data and other variables related to the subjects and controls are presented in Tables 1 and 2.

**Table 1:** Characteristics of subjects and controls.

	Participants		
Variables	Subjects (n = 442)	Controls (n = 196)	*P* value
Age (mean ± SD, yrs)	35.22 ± 4.91	32.34 ± 4.34	.000
The prevalence of smoking (%)	2.06	2.31	>.05
The prevalence of alcohol consumption (%)	14.35	15.71	>.05
The prevalence of chronic disease^∗^ (%)	10.18	10.71	>.05
The prevalence of overweight (BMI: 25–29.99) (%)	20.51	19.18	>.05
The prevalence of obese (BMI ≥ 30) (%)	8.55	10.96	>.05
The numbers of pregnancy	1.72 ± 1.10	N/A	N/A
The numbers of children	1.03 ± 0.17	N/A	N/A
Duration of infertility (yrs)	N/A	2.20 ± 1.89	N/A

**Table 2:** Clinical data and variables’ data on the ovarian reserve of subjects and controls.

		Age groups											
		Total		Group 1 (23–29 yrs)		Group 2 (30–34 yrs)		Group 3 (35–39 yrs)		Group 4 (40–49 yrs)			
Variables	Participants	Mean ± SD	2.5–97.5%	Mean ± SD	2.5–97.5%	Mean ± SD	2.5–97.5%	Mean ± SD	2.5–97.5%	Mean ± SD	2.5–97.5%	*P* value^∗^	*P* value^†^
Age (yrs)	Subjects	35.22 ± 4.91	26.00–45.70	27.38 ± 1.64	23.20–29.00	32.17 ± 1.337	30.00–34.00	36.82 ± 1.51	35.00–39.00	42.22 ± 2.21	40.00–48.00	NA	.000
	Controls	32.34 ± 4.34	25.00–43.08	27.62 ± 1.52	23.40–29.00	31.95 ± 1.41	30.00–34.00	36.16 ± 1.28	35.00–39.00	42.20 ± 2.11	40.00–46.00	NA	
The numbers of pregnancy	Subjects	1.72 ± 1.10	1.00–5.00	1.36 ± 0.76	1.00–4.00	1.42 ± 0.79	1.00–3.05	1.83 ± 1.19	1.00–5.00	2.23 ± 1.32	1.00–6.00	0.000	NA
The numbers of children	Subjects	1.03 ± 0.17	1.00–1.08	1.00 ± 0.00	1.00–1.00	1.00 ± 0.00	1.00–1.00	1.06 ± 0.26	1.00–2.00	1.03 ± 0.18	1.00–2.00	.021	NA
Duration of infertility (yrs)	Controls	2.20 ± 1.89	1.00–7.00	1.67 ± 0.97	1.00–4.70	2.10 ± 1.53	1.00–7.00	2.75 ± 2.07	1.00–8.35	3.73 ± 4.45	1.00–14.00	.002	NA
Height (centimeter)	Subjects	162.65 ± 4.24	155.00–170.00	164.75 ± 3.91	157.00–170.00	162.36 ± 4.27	155.00–171.95	160.89 ± 4.20	152.00–168.00	164.05 ± 3.53	158.00–170.00	.010	.451
	Controls	162.10 ± 5.85	149.70–173.30	161.13 ± 7.15	150.00–174.50	162.52 ± 4.60	152.00–171.80	162.25 ± 5.92	148.00–170.30	166.00 ± 8.49	160.00–169.60	.641	
Weight (kg)	Subjects	63.06 ± 12.03	46.00–95.35	72.19 ± 17.78	54.00–104.40	62.28 ± 11.75	44.05–95.00	62.43 ± 8.59	49.00–80.00	58.90 ± 7.76	47.50–74.80	.006	.636
	Controls	62.17 ± 13.57	44.55–101.05	60.08 ± 14.16	42.00 ± 100.75	58.34 ± 9.67	45.00–80.00	71.44 ± 15.29	45.00–97.20	72.50 ± 13.44	63.00–78.20	.006	
BMI	Subjects	23.81 ± 4.19	17.95–36.20	26.44 ± 5.57	19.49–36.12	23.62 ± 4.33	17.05–36.20	24.11 ± 3.16	20.20–30.26	21.87 ± 2.70	17.99–27.69	.010	.797
	Controls	23.63 ± 4.89	17.46–38.97	23.04 ± 4.55	17.48–35.30	22.10 ± 3.66	17.36–30.13	27.18 ± 6.00	17.58–38.99	26.16 ± 2.20	24.61–27.10	.004	
AFC	Subjects	10.58 ± 5.80	3.00–25.85	16.11 ± 7.27	7.00–25.00	11.68 ± 5.65	3.00–23.30	9.29 ± 4.87	3.00–22.75	6.60 ± 2.80	3.00–11.20	.000	.086
	Controls	12.44 ± 5.69	3.10–24.00	16.70 ± 3.40	12.00–21.80	11.96 ± 5.74	4.00–24.00	10.29 ± 4.96	4.00–16.20	4.50 ± 2.12	3.00–5.40	.009	
LH (IU/L)	Subjects	4.53 ± 5.74	1.04–15.84	3.80 ± 1.72	1.09–10.41	4.27 ± 3.74	1.01–12.89	4.53 ± 7.03	1.03–13.22	5.53 ± 7.93	1.22–41.94	.438	.373
	Controls	4.10 ± 2.19	1.54–9.87	4.67 ± 2.79	1.47–12.86	4.03 ± 1.73	1.44–8.51	3.59 ± 2.20	1.20–9.31	3.44 ± 1.89	1.60–7.09	.152	
FSH (IU/L)	Subjects	7.17 ± 6.84	2.45–19.77	6.21 ± 2.50	1.61–13.65	6.27 ± 6.55	2.05–10.06	6.64 ± 3.91	2.43–17.97	10.37 ± 10.94	3.31–55.84	.001	.124
	Controls	6.29 ± 2.26	2.42–12.02	5.99 ± 1.64	2.07–10.98	6.64 ± 2.54	3.65–13.64	5.99 ± 2.27	1.37–12.43	5.86 ± 2.33	1.31–10.08	.355	
TT (nmol/L)	Subjects	0.98 ± 0.39	0.46–2.09	1.11 ± 0.44	0.51–2.98	1.05 ± 0.43	0.52–2.42	0.94 ± 0.32	0.45–1.59	0.86 ± 0.25	0.43–1.69	.000	.267
	Controls	1.03 ± 0.41	0.44–2.16	1.08 ± 0.45	0.28–2.40	1.00 ± 0.37	0.38–2.04	1.08 ± 0.49	0.53–2.65	0.85 ± 0.18	0.56–1.13	.333	
FSH/LH ratio	Subjects	2.04 ± 1.23	0.41–5.20	1.91 ± 1.07	0.61–5.20	1.88 ± 1.18	0.41–4.83	2.05 ± 1.27	0.30–5.78	2.47 ± 1.25	0.40–5.93	.017	.644
	Controls	2.15 ± 3.62	0.53–44.60	1.68 ± 0.96	0.27–4.43	2.43 ± 5.14	0.59–12.95	2.18 ± 1.13	0.17–4.57	2.05 ± 1.08	0.56–3.78	.767	
E2 (pg/mL)	Subjects	56.12 ± 61.54	10.78–222.03	42.50 ± 22.27	12.10–104.00	54.70 ± 49.02	17.93–216.33	61.66 ± 55.42	11.33–244.00	64.09 ± 100.17	11.80–518.90	.333	.007
	Controls	41.93 ± 22.81	15.60–103.45	37.37 ± 13.01	13.40–77.60	41.73 ± 14.25	22.55–87.83	48.58 ± 44.58	11.00–203.25	46.10 ± 26.57	20.00–104.00	.261	
PRL (ng/mL)	Subjects	14.83 ± 9.78	5.39–49.77	18.91 ± 14.64	5.26–69.19	13.82 ± 6.45	4.41–30.64	15.92 ± 11.11	5.49–60.46	12.36 ± 8.01	5.11–46.29	.005	.271
	Controls	15.93 ± 9.78	6.29–51.08	15.51 ± 6.52	6.57–36.31	17.40 ± 12.29	6.72–65.10	12.04 ± 4.41	3.97–22.45	16.74 ± 10.02	4.04–35.24	.152	
AMH (ng/mL)	Subjects	2.533 ± 2.146	0.095–8.189	4.229 ± 3.084	0.390–13.000	3.163 ± 2.050	0.162–8.700	2.270 ± 1.700	0.224–7.508	1.032 ± 1.052	0.033–4.404	.000	.001
	Controls	3.189 ± 2.551	0.395–8.684	4.114 ± 2.620	0.619–10.648	3.235 ± 2.704	0.407–7.827	2.343 ± 1.801	0.214–7.090	1.673 ± 1.546	0.177–4.332	.001	
ORPI	Subjects	1.28 ± 1.87	0.01–6.64	4.37 ± 3.58	0.91–5.89	1.34 ± 1.39	0.02–4.07	0.74 ± 0.63	0.05–2.28	0.18 ± 0.19	0.01–0.53	.000	.141
	Controls	1.88 ± 2.68	0.04–8.53	3.34 ± 1.96	1.13–6.66	1.72 ± 3.14	0.03–4.08	0.86 ± 0.81	0.05–1.76	0.21 ± 0.25	0.04–0.31	.003	

### 3.2 A comparison of AFC, serum AMH levels, and ORPI among subgroups of subjects and controls

**Figure 1:**
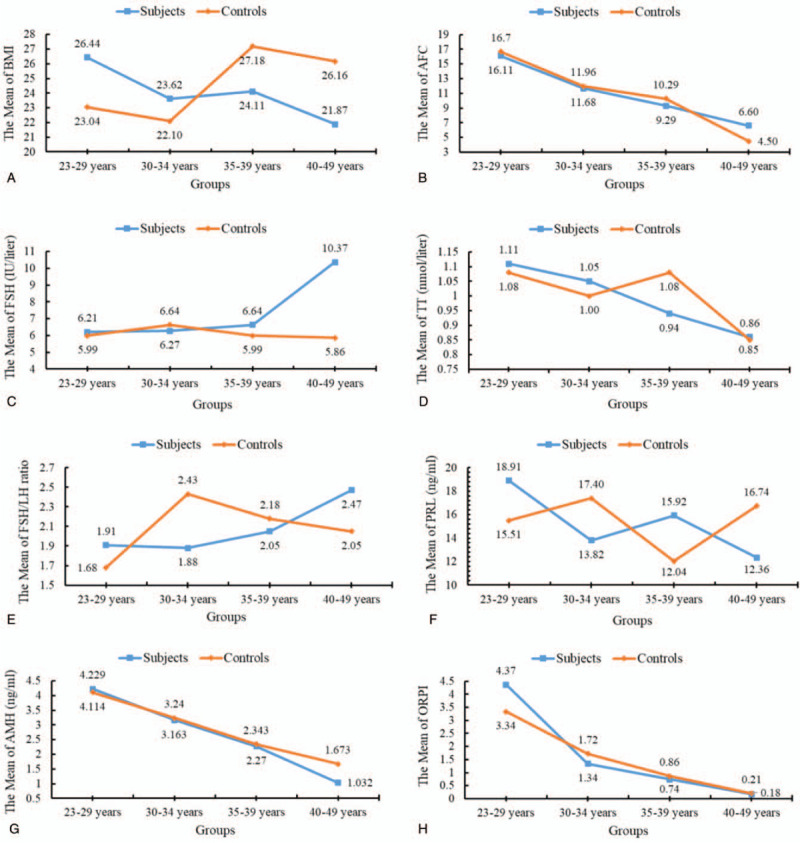
Means plots of variables presented statistical difference among age subgroups. AFC = antral follicle count, AMH = anti-Müllerian hormone, BMI = body mass index, FSH = follicle-stimulating hormone, LH = luteinizing hormone, ORPI = ovarian response prediction index, PRL = prolactin, TT = total testosterone.

For both subjects and controls, AFC, serum AMH levels, and ORPI gradually decreased with increasing age; this was the case for all subgroups (presented in Table 2 and Fig. 1(B), (G), and (H)). The range of 4 subgroups on mean AFC, mean AMH level, and mean ORPI presented in Table 2. One-way ANOVA showed that there were significant differences among the 4 age subgroups in terms of AFC (*P* < .001), AMH level (*P* < .01), ORPI (*P* < .01), weight (*P* < .01), and BMI (*P* < .05, Fig. 1(A)). For the age-related reduction of variables, the value of AFC was 0.64 AFCs/year vs 0.84 AFCs/year, and the rate of AFC was 3.98%/year vs 5.01%/year; the value of AMH was 0.215 ng/mL/year vs 0.167 ng/mL/year, and the rate of AMH was 5.09%/year vs 4.07%/year; furthermore, the value of ORPI was 0.28/year vs 0.21/year, and the rate of ORPI was 6.46%/year vs 6.43%/year, in both subjects and controls, respectively.

### 3.3 A comparison of serum FSH and TT levels, and FSH/LH ratio across subject subgroups

In the subject subgroups, serum FSH level and FSH/LH ratio gradually increased, and TT level gradually decreased, with increasing age (presented in Table 2 and Fig. 1(C)–(E)). The range of 4 subgroups on mean FSH level, FSH/LH ratio, and TT level is presented in Table 2. One-way ANOVA showed that there were significant differences among the 4 age subgroups in terms of the number of pregnancies (*P *< .001), the number of children (*P* < .05), height (*P* < .05), along with serum FSH (*P* < .01), FSH/LH ratio (*P* < .5), TT (*P* < .001), and prolactin levels (*P* < .01, Fig. 1(F)).

### 3.4 A comparison of the duration of infertility among control subgroups

In the control subgroups, the duration of infertility gradually increased with increasing age. One-way ANOVA showed that there were significant differences among the 4 age groups in terms of the duration of infertility (*P* < .01).

### 3.5 A comparison of age, serum E2, and AMH levels between subjects and controls

One-way ANOVA showed that there was significant difference in terms of age (35.22 ± 4.91 vs 32.34 ± 4.34 years, *P* < .001), serum E2 (56.12 ± 61.54 vs 41.93 ± 22.81 pg/mL, *P* < .01), and AMH levels (2.533 ± 2.146 vs 3.189 ± 2.551 ng/mL, *P* < .01) between subjects and controls.

### 3.6 The application of multivariate analysis of covariance (ANCOVA/MANCOVA) to control for confounding factors and to differentiate influential factors

In order to exclude factors that may influence the status of ovarian reserve, particularly those that may have differed between subjects and controls, we used ANCOVA/MANCOVA to control for confounding factors and analyzed differences between subjects and controls.

ANCOVA/MANCOVA showed there was a significant multivariate linear regression relationship between age, AFC, FSH, and AMH (*F* = 33.369, *P* < .001). After controlling for confounding factors (age, AFC, and FSH), there was no significant difference in serum AMH level between subjects and controls (*F* = 0.969, *P* > .05).

ANCOVA/MANCOVA showed there was a significant multivariate linear regression relationship between TT, AMH, and AFC (*F* = 6.724, *P* < .001). After controlling for confounding factors (TT and AMH), there was no significant difference in AFC between subjects and controls (*F* = 2.250, *P* > .05).

ANCOVA/MANCOVA showed there was a significant multivariate linear regression relationship between BMI, LH, FSH/LH ratio, E2, AMH, and FSH (*F* = 7.556, *P* < .001). After controlling for confounding factors (BMI, LH, FSH/LH ratio, E2, and AMH), there was no significant difference in serum FSH level between subjects and controls (*F* = 0.425, *P* > .05).

ANCOVA/MANCOVA showed there was a significant multivariate linear regression relationship between LH, FSH, and E2 (*F* = 6.430, *P* < .001). After controlling for confounding factors (LH and FSH), there was no significant difference in E2 between subjects and controls (*F* = 0.960, *P* > .05).

### 3.7 Pearson's correlation analysis between variables relating to ovarian reserve

Pearson's correlation analysis for both subjects and controls, indicated that there were positive correlations between AMH and AFC (*P* < .001); moreover, there were negative correlations between age and AFC (*P* < .001), AMH (*P* < .001), ORPI (*P* < .001, *P* < .05), and between FSH and AFC (*P* < .05), E2 (*P* < .05, *P* < .01), and AMH (*P* < .001).

Pearson's correlation analysis for the subjects showed that there were positive correlations between age and FSH (*P* < .001), FSH/LH ratio (*P* < .01), between BMI and AFC (*P* < .05), and between ORPI and AFC (*P* < .001), TT (*P* < .001), and AMH (*P* < .001); however, there were negative correlations between FSH/LH ratio and AFC (*P* < .01), and AMH (*P* < .001).

### 3.8 The comparison and AUC of ROC curves on variables evaluated ovarian reserve

**Figure 2:**
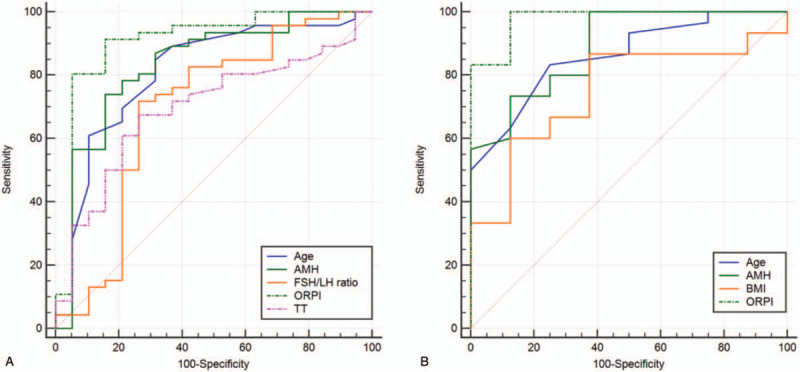
The comparison on ROC curves of variables evaluated ovarian reserve. (A) ROC curves of subjects’ variables. (B) ROC curves of controls’ variables. AMH = anti-Müllerian hormone, AUC = the area under the curve, BMI = body mass index, FSH = follicle-stimulating hormone, LH = luteinizing hormone, ORPI = ovarian response prediction index, ROC curve = receiver operating characteristic curve, TT = total testosterone.

In general, AFC < 7 was one of the standards indicating decline of ovarian reserve.^[10]^ Using the abovementioned standards and the ROC curve to evaluate the significant variables of ovarian reserve decrease, the variables of subjects included age, AMH, FSH/LH ratio, ORPI, and TT, the corresponding AUC was 0.731, 0.817, 0.688, 0.902, and 0.700, respectively; furthermore, the variables of controls included age, AMH, BMI, and ORPI, the corresponding AUC was 0.8000, 0.865, 0.750, and 0.986, respectively. In terms of evaluation of ovarian reserve, ORPI was more valuable than other variables. When the variables were used to evaluate the ovarian reserve, the cut-off values of age (33 vs 38 years), AMH (2.22 vs 2.49 ng/mL), and ORPI (0.45 vs 0.53) from controls were less than the values from subjects. AUC, cut-off values, sensitivity, and specificity of variables evaluated ovarian reserve were presented in Table 3 and Figure 2.

**Table 3:** The ROC curve AUC and cut-off values of variables on evaluating ovarian reserve.

		AUC^∗^				
Participants	Variables	Value (95% confidence interval)	*P* value	Cut-off value	Sensitivity (%)	Specificity (%)
Subjects	Age	0.731 (0.624, 0.821)	.0002	≤38	87.72	53.57
	AMH	0.817 (0.718, 0.892)	<.0001	>2.49	66.67	85.71
	FSH/LH ratio	0.688 (0.565, 0.794)	.0162	≤1.82	68.75	76.19
	ORPI	0.902 (0.818, 0.956)	<.0001	>0.53	80.36	92.86
	TT	0.700 (0.575, 0.807)	.0038	>0.91	68.09	73.68
Controls	Age	0.800 (0.650, 0.906)	.0001	≤33	84.37	63.64
	AMH	0.865 (0.726, 0.950)	<.0001	>2.22	75.00	81.82
	BMI	0.750 (0.583, 0.876)	.0063	≤23.44	86.67	62.50
	ORPI	0.986 (0.892, 1.000)	<.0001	>0.45	100.00	90.91

According to our results, ovarian reserve would decrease when subjects’ age and FSH/LH ratio, and controls’ age and BMI were higher than the cut-off values; on the contrary, ovarian reserve presented similar phenomena when the AMH, ORPI, and TT levels of subjects, and AMH and ORPI of controls were less than the cut-off values.

## 4 Discussion

AMH is known to be one of the most stable and reliable laboratory markers for the assessment of ovarian reserve, and is known to be a key variable for ovarian reserve-related outcomes and clinical practice, and has been widely used in clinical practice.^[11]^ However, the number of AFCs determined can depend upon the quality of the ultrasonic equipment used, and is known to vary among different sonographers.

Although many studies on serum AMH level have been carried out, there is some controversy with regards to the reference values for AMH in the healthy female population. This is because previous studies used different measurement methods and reagents, and analyzed different samples from different populations or different ethnicities. A previous study^[12]^ involving female participants aged 25 to 35 years, reported serum AMH concentrations for fertile control, normal ovarian reserve (NOR), and diminished ovarian reserve groups was 2.0 ± 0.6 ng/mL, 1.9 ± 0.16ng/mL, and 0.89 ± 0.47 ng/mL, respectively; there was a significant difference between the fertile control, NOR, and diminished ovarian reserve groups (*P* = .001). Another prospective study^[13]^ indicated that the mean serum level of AMH in infertile women aged 24 to 48 years on day 3 of the menstrual cycle was 2.84 ± 1.57 ng/mL. Our current results showed that the AMH levels of subjects and controls were higher than that reported in previous papers. However, our AFC values fell between those reported by 2 previous studies; 1 reported AFCs of 14.68 ± 4.2,^[13]^ while another reported fertile group AFCs of 8.4 ± 4.9, and infertile group AFCs of 8.5 ± 5.3; further analysis showed that AFC and AMH did not differ between the fertile and infertile groups with regards to different age categories.^[4]^

According to the published literature, there is a general consensus of opinion with regards to age-related trends in AFC and serum AMH level in healthy women. Data have further revealed that AFC, AMH level, along with median and mean AMH levels, decrease steadily and gradually with increasing age,^[14–16]^ thus reflecting a decline of the non-growing follicle pool.^[1]^ Furthermore, the age-specific reduction of the ovarian reserve was similar in both infertile and fertile female patients; serum AMH concentration decreased by 6% or 6.2%, and AFC declined by 4.5% per year with increased age. Aged patients (36–39 years) had a 5.3% higher risk ratio of having an AMH level < 0.7 ng/mL than younger age groups (*P* < .01), and the reduction in AMH accelerated at around 25, 35, and 40 years.^[17,18]^ Another study showed that by the age of 32 years, over 50% of women had AMH levels categorized as “low fertility” (AMH ≤ 19.5 pmol/L), increasing to 75% by age 39, and a reduction in mean AMH of 1.72 pmol/L/year.^[19]^ Although it is well established that female fertility declines with age, the rate and timing of this decline varies significantly among women. This is mainly due to inter-individual differences in ovarian reserve.^[2]^ Our results indicated that for both subjects and controls, AFC and serum AMH levels had similar age-related trends to those described in the previous literature, although the value and rate of decline for both variables, excluding the AFC rate in controls, was less than that described in previous studies.

With regards to the subjects, we found that the serum FSH and FSH/LH levels of fertile women increased, and both serum TT level and ORPI decreased with increasing age; there were significant differences among the 4 age subgroups. However, with regards to the controls, these 4 variables showed no trend and differences with increasing age. We found that AFC, serum AMH levels, and ORPI were stable and reliable in both the fertile and infertile groups, and could more accurately reflect the ovarian reserve. In addition, our ROC curve analysis results identified that age, AMH, and ORPI were significant variables of subjects and controls for evaluating ovarian reserve, the cut-off value of age was similar to the above-mentioned report.^[19]^ However, we found that FSH, FSH/LH ratio, and TT were more easily changed by influential factors, particularly in infertile patients. We consider that the AMH results in our subjects could represent the real status of fertile women; this is because our healthy subjects had experienced natural pregnancies and childbirth. Furthermore, the commercial reagents we used (acquired from Roche Diagnostics GmbH) were sensitive and reliable; when combined with an electrochemiluminescence immunoassay, these reagents provided an efficient assay for serum AMH levels.

The existing literature shows significant controversy with regards to variables related to fertile and infertile women. AFC, the median, and mean AMH concentrations, were significantly lower, but mean FSH serum levels were higher in the infertility group than the control group; however, this reduction was greater in the infertile group; mean LH levels were not consistent.^[8,15]^ A previous study reported a difference in mean AMH (2.53 ± 1.90 ng/mL vs 3.54 ± 1.42ng/mL), AFC, and ovarian volume between the nulliparous and multiparous group (*P* < .001), although FSH levels did not differ significantly.^[6]^ In another study, significant differences were detected between all ovarian reserve groups with regards to AMH, E2, and FSH (*P* < .001 for all).^[20]^ However, infertile patients had similar AMH levels, AFC, AMH/AFC ovarian indices, and the prevalence of very low AMH levels (<5 pmol/L) compared with controls in an age-adjusted linear regression analysis, or after controlling for age, race, BMI, smoking history, and study site.^[3,5]^ Without the use of multivariate analysis of variance, some published papers still found no difference in ovarian reserve when compared between fertile and infertile women. Furthermore, AFC and AMH levels did not differ when compared between fertile and infertile populations from all age categories, and the reduction in ovarian reserve of the infertile patients was directly related to age, not infertility.^[4,21]^ One previous study showed that biomarkers indicating diminished ovarian reserve, as compared with normal ovarian reserve, were not associated with reduced fertility; these findings did not support the use of FSH or AMH levels to assess natural fertility in women.^[22]^ Our current results showed that there were significant differences in age, serum E2, and AMH levels (*P* < .01 for all) between subjects and controls; however, when we controlled for confounding factors (age, BMI, TT, and LH), we found there were no differences in serum AMH, FSH, E2 levels, or AFC, when compared between subjects and controls (*P* > .05 for all). This indicates that diminished ovarian reserve is a manifestation of female aging, and that fertile women particularly conform to the natural aging rule; furthermore, the ovarian reserve of infertile women may be influenced by other concurrent diseases.

This study indicated, for both subjects and controls, there were positive correlations between AMH and AFC, and that there were negative correlations between age and AFC, AMH, and ORPI, and between FSH and AFC, E2, and AMH. Moreover, several correlations only presented in the subject group, including positive correlations between age and FSH, FSH/LH, and between BMI and AFC, and between ORPI and AFC, TT, and AMH; negative correlations were observed between FSH/LH ratio and AFC, and AMH. Importantly, there was a specific phenomenon that the trends for change with aging described above, and the correlations between several variables, only presented in subjects. These findings were not evident in the control group, potentially illustrating that the diminished ovarian reserve of fertile women conformed to the process of natural aging, and could be reflected by correlations between certain variables. The correlations we identified in the present study agreed with the published literature,^[4,13,23]^ in that a significant correlation was observed between AMH, or age and FSH serum levels, and AFC, and between AMH and age. Furthermore, AMH was positively correlated with E2, and the free androgen index during adolescence, as well as during the reproductive phases.^[24]^ A positive correlation between AMH and testosterone has also been reported, although controversy persists.^[25,26]^ Women of reproductive age with obesity were previously reported to have AMH concentrations that were 23.7% lower than those with a BMI ≤ 25 kg/m^2^ (2.9 ng/mL vs 3.8 ng/mL); BMI was inversely associated with AMH.^[27]^ However, other studies have stated that AMH has no correlation, or a positive correlation, with BMI.^[24,28,29]^

Ethnicity could cause effect on AMH levels.^[28,30]^ One limitation of our study was that we were not able to explore the influence of ethnicity.

## 5 Conclusions

For both subjects and controls, AFC, AMH levels, and ORPI decreased gradually with increasing age, and 3 variables presented similar age-related trends. There were positive correlations between AMH and AFC, and negative correlations between age and AFC, AMH and ORPI. Although there were significant differences in age, serum E2, and AMH levels between subjects and controls, we controlled for confounding factors (age, BMI, TT, and LH) and found that there were no differences in serum AMH, FSH, E2 levels, and AFC, when compared between subjects and controls. The significant variables of subjects and controls for evaluating ovarian reserve included age, AMH and ORPI, and ORPI was more valuable than other variables. In conclusion, diminished ovarian reserve represents a manifestation of aging, and can be affected by several factors. Finally, we found that there was no difference in ovarian reserve when compared between fertile and infertile women, and that there was no correlation with infertility.

## Acknowledgments

Authors would like to acknowledge the medical team of Reproductive Medicine Center of Peking University International Hospital (PKUIH), and Dr Jie Zhang, Dr Xin-Ping Sun and Dr Ming Yang of Clinical Laboratory Department of PKUIH. And we gratefully acknowledge the help of Dr Dian He for the statistical analysis. Dr He was from Department of Epidemiology and Health Statistics, School of Public Health, Capital Medical University, Beijing, China. We also thank International Science Editing (http://www.internationalscienceediting.com) for editing this manuscript.

## Author contributions

Shan-Jie Zhou mainly interpreted the data, conducted integrative analysis, and prepared the manuscript as a major contributor. Shan-Jie Zhou, Tie-Cheng Sun, Ling-Li Song, and Li Tian collected the data and performed the statistical analysis. Ming Yang and Xing-Ping Sun contributed to measure the concentrations of reproductive hormones and AMH. Li Tian contributed to measure the number of AFCs. Shan-Jie Zhou and Li Tian designed the study and revised the manuscript. All authors read and approved the final manuscript.

**Conceptualization:** Shan-Jie Zhou, Tie-Cheng Sun, Li Tian.

**Data curation:** Shan-Jie Zhou, Ling-Li Song, Ming Yang, Xin-Ping Sun, Li Tian.

**Formal analysis:** Shan-Jie Zhou, Tie-Cheng Sun, Ling-Li Song, Ming Yang, Xin-Ping Sun, Li Tian.

**Funding acquisition:** Shan-Jie Zhou.

**Investigation:** Shan-Jie Zhou, Tie-Cheng Sun, Ling-Li Song, Ming Yang, Xin-Ping Sun.

**Methodology:** Shan-Jie Zhou, Ming Yang, Li Tian.

**Project administration:** Shan-Jie Zhou, Tie-Cheng Sun, Ling-Li Song, Li Tian.

**Resources:** Shan-Jie Zhou, Xin-Ping Sun.

**Supervision:** Li Tian.

**Validation:** Shan-Jie Zhou, Tie-Cheng Sun, Ming Yang.

**Visualization:** Shan-Jie Zhou, Tie-Cheng Sun.

**Writing – original draft:** Shan-Jie Zhou, Tie-Cheng Sun, Li Tian.

**Writing – review & editing:** Shan-Jie Zhou, Li Tian.

## References

[1] Dillon KE Gracia CR. What is normal ovarian reserve? Semin Reprod Med 2013;31:427–36. 24101223 10.1055/s-0033-1356478

[2] Broer SL Broekmans FJ Laven JS. Anti-Mullerian hormone: ovarian reserve testing and its potential clinical implications. Hum Reprod Update 2014;20:688–701. 24821925 10.1093/humupd/dmu020

[3] Hvidman HW Bentzen JG Thuesen LL. Infertile women below the age of 40 have similar anti-Mullerian hormone levels and antral follicle count compared with women of the same age with no history of infertility. Hum Reprod 2016;31:1034–45. 26965431 10.1093/humrep/dew032

[4] Bozkurt B Erdem M Mutlu MF. Comparison of age-related changes in anti-Mullerian hormone levels and other ovarian reserve tests between healthy fertile and infertile population. Hum Fertil (Camb) 2016;19:192–8. 27499425 10.1080/14647273.2016.1217431

[5] Greenwood EA Cedars MI Santoro N. Antimullerian hormone levels and antral follicle counts are not reduced compared with community controls in patients with rigorously defined unexplained infertility. Fertil Steril 2017;108:1070–7. 29202959 10.1016/j.fertnstert.2017.09.015

[6] Moini A Hedayatshodeh M Hosseini R. Association between parity and ovarian reserve in reproductive age women. Eur J Obstet Gynecol Reprod Biol 2016;207:184–7. 27865943 10.1016/j.ejogrb.2016.10.024

[7] Okunola T Olusegun Ajenifuja K Morebise Loto O. Follicle stimulating hormone and anti-mullerian hormone among fertile and infertile women in Ile-Ife, Nigeria: is there a difference? Int J Fertil Steril 2017;11:33–9. 28367303 10.22074/ijfs.2016.4645 PMC5215709

[8] Yucel B Kelekci S Demirel E. Decline in ovarian reserve may be an undiagnosed reason for unexplained infertility: a cohort study. Arch Med Sci 2018;14:527–31. 29765438 10.5114/aoms.2016.58843 PMC5949901

[9] Oliveira JB Baruffi RL Petersen CG. A new ovarian response prediction index (ORPI): implications for individualised controlled ovarian stimulation. Reprod Biol Endocrinol 2012;10:94. 23171004 10.1186/1477-7827-10-94 PMC3566907

[10] Ferraretti AP La Marca A Fauser BC. ESHRE consensus on the definition of ’poor response’ to ovarian stimulation for in vitro fertilization: the Bologna criteria. Hum Reprod 2011;26:1616–24. 21505041 10.1093/humrep/der092

[11] Tobler KJ Shoham G Christianson MS. Use of anti-mullerian hormone for testing ovarian reserve: a survey of 796 infertility clinics worldwide. J Assist Reprod Genet 2015;32:1441–8. 26347341 10.1007/s10815-015-0562-7 PMC4615913

[12] Parveen N Rehman DE Jawed S. Comparison of serum anti-mullerian hormone among fertile and infertile normal and diminished ovarian reserve groups. J Pak Med Assoc 2016;66:1060–3. 27654719

[13] Scheffer JAB Scheffer B Scheffer R. Are age and anti-Mullerian hormone good predictors of ovarian reserve and response in women undergoing IVF? JBRA Assist Reprod 2018;22:215–20. 29949322 10.5935/1518-0557.20180043 PMC6106624

[14] Seifer DB Baker VL Leader B. Age-specific serum anti-Mullerian hormone values for 17,120 women presenting to fertility centers within the United States. Fertil Steril 2011;95:747–50. 21074758 10.1016/j.fertnstert.2010.10.011

[15] Raeissi A Torki A Moradi A. Age-specific serum anti-mullerian hormone and follicle stimulating hormone concentrations in infertile Iranian women. Int J Fertil Steril 2015;9:27–32. 25918589 10.22074/ijfs.2015.4205 PMC4410034

[16] Woloszynek RR Brito LP Batista MC. Validation of an immunoassay for anti-Mullerian hormone measurements and reference intervals in healthy Brazilian subjects. Ann Clin Biochem 2015;52:67–75. 25249661 10.1177/0004563214554462

[17] Khan HL Bhatti S Suhail S. Antral follicle count (AFC) and serum anti-Mullerian hormone (AMH) are the predictors of natural fecundability have similar trends irrespective of fertility status and menstrual characteristics among fertile and infertile women below the age of 40 years. Reprod Biol Endocrinol 2019;17:20. 30744650 10.1186/s12958-019-0464-0 PMC6371573

[18] Zhu J Li T Xing W. Chronological age vs biological age: a retrospective analysis on age-specific serum anti-Mullerian hormone levels for 3280 females in reproductive center clinic. Gynecol Endocrinol 2018;34:890–4. 29671678 10.1080/09513590.2018.1462317

[19] Naasan MN Harrity C Pentony L. Anti-Mullerian hormone normogram in an Irish subfertile population. Ir J Med Sci 2015;184:213–8. 24563261 10.1007/s11845-014-1089-0

[20] Gorkem U Kucukler FK Togrul C. Obesity does not compromise ovarian reserve markers in infertile women. Geburtshilfe Frauenheilkd 2019;79:79–85. 30686837 10.1055/a-0650-4723 PMC6336460

[21] Somigliana E Lattuada D Colciaghi B. Serum anti-Mullerian hormone in subfertile women. Acta Obstet Gynecol Scand 2015;94:1307–12. 26332870 10.1111/aogs.12761

[22] Steiner AZ Pritchard D Stanczyk FZ. Association between biomarkers of ovarian reserve and infertility among older women of reproductive age. JAMA 2017;318:1367–76. 29049585 10.1001/jama.2017.14588 PMC5744252

[23] Barbakadze L Kristesashvili J Khonelidze N. The correlations of anti-mullerian hormone, follicle-stimulating hormone and antral follicle count in different age groups of infertile women. Int J Fertil Steril 2015;8:393–8. 25780521 10.22074/ijfs.2015.4179 PMC4355926

[24] Cui L Qin Y Gao X. Antimullerian hormone: correlation with age and androgenic and metabolic factors in women from birth to postmenopause. Fertil Steril 2016;105:481–5e1. 26549157 10.1016/j.fertnstert.2015.10.017

[25] Ikeda K Baba T Morishita M. Long-term treatment with dehydroepiandrosterone may lead to follicular atresia through interaction with anti-Mullerian hormone. J Ovarian Res 2014;7:46. 24851135 10.1186/1757-2215-7-46 PMC4029985

[26] Rodrigues JK Navarro PA Zelinski MB. Direct actions of androgens on the survival, growth and secretion of steroids and anti-Mullerian hormone by individual macaque follicles during three-dimensional culture. Hum Reprod 2015;30:664–74. 25567619 10.1093/humrep/deu335 PMC4325670

[27] Bernardi LA Carnethon MR de Chavez PJ. Relationship between obesity and anti-Mullerian hormone in reproductive-aged African American women. Obesity (Silver Spring) 2017;25:229–35. 27925445 10.1002/oby.21681 PMC5182136

[28] Simoes-Pereira J Nunes J Aguiar A. Influence of body mass index in anti-Mullerian hormone levels in 951 non-polycystic ovarian syndrome women followed at a reproductive medicine unit. Endocrine 2018;61:144–8. 29470775 10.1007/s12020-018-1555-y

[29] Albu D Albu A. The relationship between anti-Mullerian hormone serum level and body mass index in a large cohort of infertile patients. Endocrine 2019;63:157–63. 30238328 10.1007/s12020-018-1756-4

[30] Moy V Jindal S Lieman H. Obesity adversely affects serum anti-mullerian hormone (AMH) levels in Caucasian women. J Assist Reprod Genet 2015;32:1305–11. 26194744 10.1007/s10815-015-0538-7 PMC4595398

